# From Rat to Human: Regulation of Renin-Angiotensin System Genes by Sry

**DOI:** 10.1155/2012/724240

**Published:** 2012-01-22

**Authors:** Jeremy W. Prokop, Ingrid Kazue Mizuno Watanabe, Monte E. Turner, Adam C. Underwood, Almir S. Martins, Amy Milsted

**Affiliations:** ^1^Department of Biology and Integrated Bioscience Ph.D. Program, The University of Akron, Akron, OH 44325-3908, USA; ^2^Nephrology Division, Department of Medicine, Federal University of Sao Paulo, Sao Paulo, SP, Brazil; ^3^Division of Mathematics and Sciences, Walsh University, North Canton, OH 44720, USA; ^4^Department of Physiology and Biophysics, ICB, Federal University of Minas Gerais, 31270-010 Belo Horizonte, MG, Brazil

## Abstract

The testis determining protein, Sry, has functions outside of testis determination. Multiple Sry loci are found on the Y-chromosome. Proteins from these loci have differential activity on promoters of renin-angiotensin system genes, possibly contributing to elevation of blood pressure. Variation at amino acid 76 accounts for the majority of differential effects by rat proteins Sry1 and Sry3. Human SRY regulated rat promoters in the same manner as rat Sry, elevating *Agt, Ren*, and *Ace* promoter activity while downregulating *Ace 2*. Human SRY significantly regulated human promoters of *AGT, REN, ACE2, AT2,* and *MAS* compared to control levels, elevating *AGT* and *REN* promoter activity while decreasing *ACE2, AT2,* and *MAS*. While the effect of human SRY on individual genes is often modest, we show that many different genes participating in the renin-angiotensin system can be affected by SRY, apparently in coordinated fashion, to produce more Ang II and less Ang-(1–7).

## 1. Introduction

Many genes on the Y chromosome that are expressed in tissues not involved in testis formation could contribute to sex differences in blood pressure and other disease phenotypes. *Sry* is believed to have evolved from the X chromosome gene *Sox3* during the process of Y-chromosome formation in therian mammals [1]. Since Sox3 has functions other than testis determination [2], *Sry* may also have additional functions outside testis determination. Sry and other Sox proteins are architectural transcription factors that bind to the minor groove of DNA, changing gene regulation through inducing a bend in the DNA [3].

**Figure 1:**
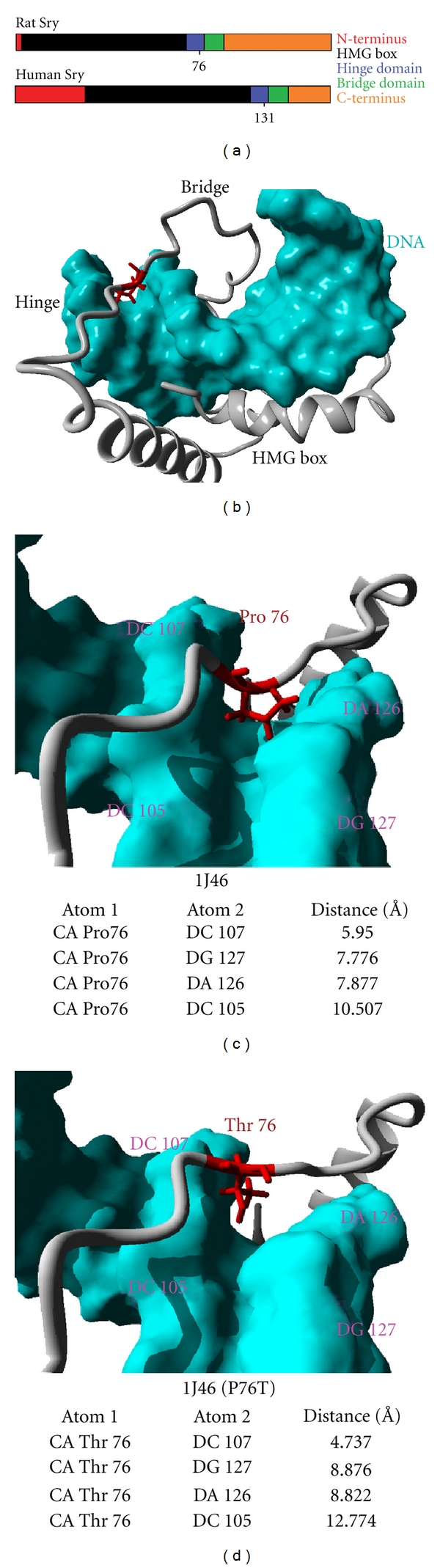
Modeling Sry variations of Sry1, Sry3, and hSRY. (a) Schematic of the rat Sry and human SRY proteins with amino acid 76 of rat (131 of human) shown. In red is the N-terminus, black the HMG box, blue the hinge domain, green the bridge domain, and orange the C-terminus. (b) Amino acid 76 (red) can be seen to interact with DNA (cyan) on modeled rat Sry1. (c) 1j46 (human SRY bound to DNA) was energy-minimized and the distances of the carbon alpha of the proline, amino acid at 76 (131), were measured from four different phosphates of DNA (DC 105, DC 107, DG 127, and DA 126). (d) 1j46 (P76T), in which the proline was substituted with a threonine, was energy-minimized and distance-measured.

The spontaneously hypertensive rat (SHR) has at least seven expressed *Sry* loci whereas the normotensive Wistar Kyoto (WKY) rat has at least six [4], lacking *Sry3*. Sry transcripts have been observed in adult rat tissues consistent with blood pressure regulation [5], and one or more of the* Sry* loci play a role in the development of hypertension in SHR [6]. Many rodent species have multiple *Sry* loci, while human and mouse have only one known locus. Sry proteins in human, rat, and other placental mammals have a homologous HMG box, hinge region, and bridge domain, with little homology in the N and C terminal ends (Figure 1(a)). The role of Sry regulation of blood pressure in humans has not been studied directly. However, with the high degree of conservation in the DNA-binding domain between human and rat, functions of Sry seen in rat are likely to translate into clinical relevance for human male blood pressure regulation.

Of the rat Sry proteins we have examined, Sry3 proteins have the largest effect on promoters of the renin-angiotensin system (RAS) genes [7]. Additionally, the Sry3 locus is found only in SHR, making it a prime candidate for elevating blood pressure. Sry1 [8] and Sry3 [9] proteins of rat have been shown to elevate blood pressure in normotensive rats. Delivery of Sry1 is known to regulate tyrosine hydroxylase promoter activity in cultured cells [10] and in rats [9]. Two amino acid differences are found between Sry1 and Sry3, a histidine (Sry1) to glutamine (Sry3) at amino acid 38 and a proline (Sry1) to threonine (Sry3) at amino acid 76 [4]. At these two amino acids, humans share one with each rat Sry protein: proline at amino acid 131 is the same as amino acid 76 of Sry1 and glutamine at amino acid 93 is the same as amino acid 38 of Sry3. The aim of this study was to identify the role of these two amino acid differences in rat, and to address the role of human SRY on regulation of rat and human RAS promoter constructs identifying possible conserved functions of Sry in blood pressure regulation across species.

## 2. Methods

### 2.1. Sry Modeling

Models for Sry1, Sry3 and hSRY proteins were created with iTASSER [11]. Models that contained a DNA-binding cleft with the best confidence score were selected. Manipulations and highlighting of structures were performed with YASARA (http://www.yasara.org/). Models were superposed to DNA using PDB structure 1j46 [12]. 1j46 is a structure determined through NMR of the human SRY HMG box bound to a fragment of DNA containing the SRY binding element. To produce structure 1j46 P76T, proline 76 was swapped with a threonine using YASARA Structure. Both 1j46 and 1j46 (P76T) were energy minimized with AMBER03 force field [13].

### 2.2. Cloning

Mutant Sry constructs were designed using pEF-1 expression vectors containing rat *Sry1* (EU984075), *Sry3* (EU984077), or human *SRY* (NM_003140) through primer directed mutagenesis. Constructs are shown in Table 1, identifying the amino acid found at 76 (131 of human). Primers were phosphorylated with T4 polynucleotide kinase (Fermentas) and used in PCR with Phusion Hot Start Taq (Finnzymes). T4 DNA ligase (Fermentas) was used for ligations. Following transformation into TAM-1 competent *E. coli* (Active Motif), clones were sequenced with BigDye Terminator chemistry on ABI 3130xl genetic analyzer (Applied Biosystems). Luciferase reporter vectors (pGL3) for rat *angiotensinogen* (*Agt), renin (Ren), angiotensin-I converting enzyme (Ace)*, and *angiotensin-2 converting enzyme* (*Ace2)* were previously described [6]. Human promoters of *AGT, REN, ACE*, *ACE 2, MAS1 oncogene *(*MAS*), and *angiotensin II subtype 2 receptor* (*AT2*) were cloned into pGL3 using primers and restriction enzymes listed in Table 2.

Because of the way the constructs were made and the way the luciferase assays were carried out, the levels of induction on these promoters can only be compared to the control for each promoter. Levels of activity on one promoter cannot be compared to those of another promoter construct.

### 2.3. Cotransfections

CHO-K1 cells (ATCC) were plated at 5 × 10^4^ cells per well into 24 well plates (COSTAR) with HAMs F12K media (Sigma) supplemented with 10% fetal bovine serum (Atlanta Biologicals), 10 mmol/L HEPES, and 30 mmol/L sodium bicarbonate and cultured in 5% CO_2_ and 95% humidity. Twenty four hours after plating, cotransfections were performed using 50 ng pEF-1 effector vector, 500 ng pGL3 reporter vector, 500 pg phRL-null Renilla control vector, 2 *μ*L TurboFect (Fermentas), and serum-free HAMs-F12K media to 100 *μ*L. After twenty four hours, cells were lysed and light intensity measured with Dual-Luciferase Reporter Assay System (Promega).

### 2.4. Statistics

Results for each promoter were analyzed with JMP by comparing relative intensity to an empty pEF-1 control vector or respective nonmutated vector. All ANOVAs that showed significance (*P* ≤ 0.05) were followed by Students *t*-tests. For all luciferase assays *n* = 3–5, each *n* represents an individual experiment of dual measurements from two duplicate wells, and error bars are standard error of the mean (SEM) and *P ≤ 0.05, **P ≤ 0.01, ***P ≤ 0.001.

## 3. Results

Models of variations at amino acids 38 and 76 previously described were created to verify whether the variation could potentially alter DNA interaction. This was determined by either modeling the protein backbone independent of DNA interaction with iTASSER or swapping amino acids in the known hSRY structure (1j46) of Sry-DNA interaction. When Sry is interacting with DNA, amino acid 38 (found in the HMG box) is found on the opposite side of the DNA interaction of the HMG box. Therefore, it does not appear to contribute to DNA interaction. Amino acid 76 of rat Sry (131 of human) is in the hinge domain (Figure 1(b) red) and interacts with DNA. A proline at amino acid 76 interacts with the DNA minor groove base pairs in a non-DNA sequence-specific manner [12]. Models of Sry3, containing a threonine at amino acid 76, show an altered protein backbone, which would change the interaction of amino acid 76 from the minor groove to the phosphate backbone of DNA (data not shown).

Exchanging the proline of hSRY 1j46 (Figure 1(c)) for a threonine (Figure 1(d)) and energy minimizing the protein bound to DNA led to the same shift in DNA interaction as seen with modeling approaches. Bond distances between the alpha carbon (CA, atom one) of either the proline (Figure 1(c)) or threonine (Figure 1(d)) with four different phosphates of DNA bases (atom two) confirm the shift relative to DNA. The CA of threonine (Figure 1(d)) is shifted closer (by 1.213 Å) to the phosphate of DC 107 (4.737 Å compared to 5.950 Å), while farther from DG127 (by 1.100 Å), DA126 (by 0.945 Å), and DC105 (by 2.267 Å) compared to proline (Figure 1(c)). This suggests the CA is shifting out of the minor groove and closer to the phosphate backbone when changed to threonine.

**Figure 2:**
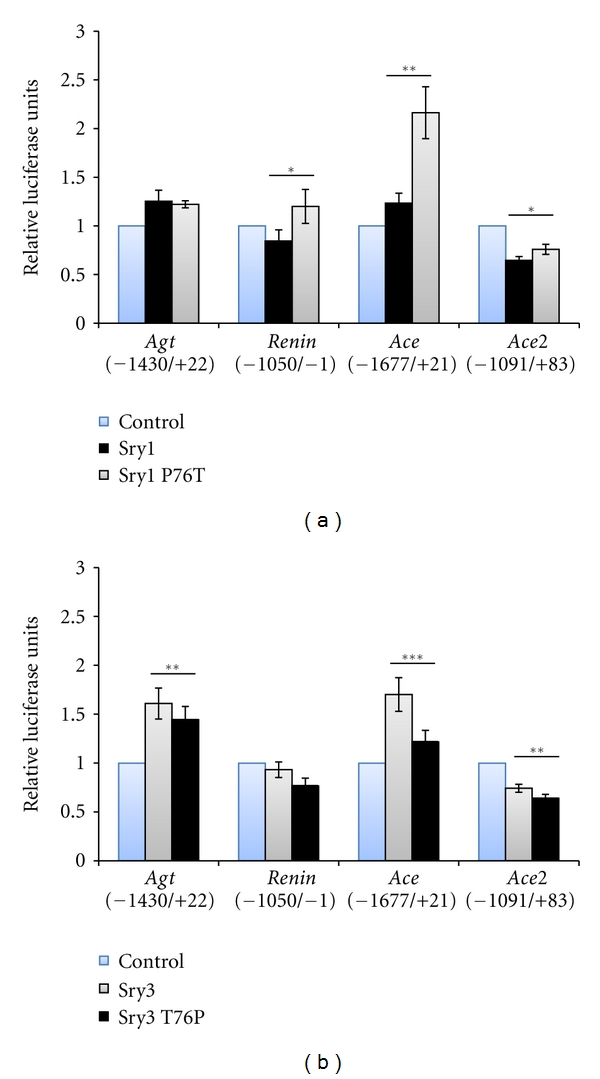
Variation at amino acid 76 of rat Sry proteins effects on rat RAS gene promoters. Amino acid variation between Sry1 and Sry3 results in differential activity on promoters of the RAS with Sry1 P76T (a) and Sry3 T76P (b). (a) Sry1 P76T significantly increased activity on *Ren, Ace*, and *Ace2*. (b) Sry3 T76P significantly decreased activity on *Agt*, *Ace*, and *Ace2*. Asterisks indicate significance from respective nonmutant Sry. *P ≤ 0.05, **P ≤ 0.01, ***P ≤ 0.001. Blue bars are the control pEF-1 vector and black bars represent proteins with a proline at amino acid 76.

**Figure 3:**
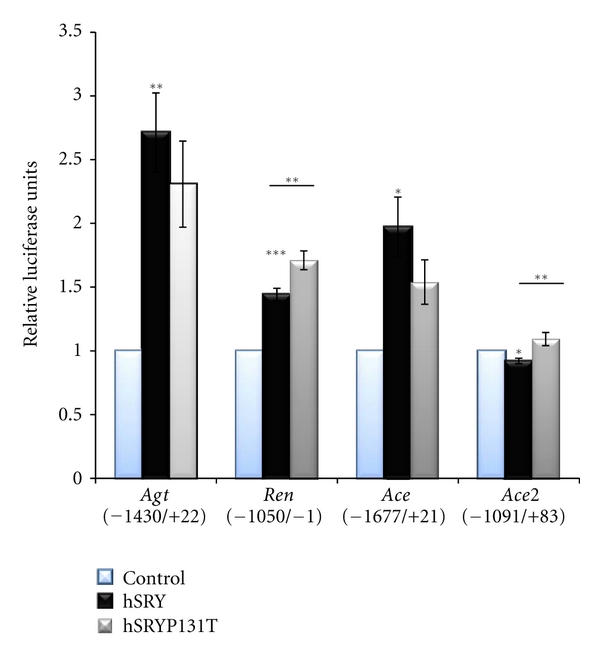
Human SRY regulation of rat RAS gene promoters. Human SRY (black) increases promoter activity of rat *Agt, Ren*, and *Ace* while decreasing *Ace2* as previously seen with Sry1 and Sry3. Mutations to the proline 131 of hSRY to threonine (gray) significantly increased activity in *Ren* and *Ace2 *while they had a trend in decreasing activity on *Agt* and *Ace*. Significance of hSRY is based on comparison to the control vector (blue) or for hSRY P131T to the hSRY values. *P ≤ 0.05, **P ≤ 0.01, ***P ≤ 0.001.

Mutation of amino acid 76 from proline to threonine in Sry1 (Sry1 P76T) significantly increased promoter activity from *Ren, Ace *and *Ace2* relative to nonmutated Sry1 (*P* ≤ 0.05, Figure 2(a)). Mutation of Sry3 at amino 76 from threonine to proline (Sry3 T76P) decreased *Agt*, *Ace*, and* Ace2* promoter activity (*P* ≤ 0.05, Figure 2(b)). Mutagenesis of rat amino acid 38 (93 of human) showed no significant effect on activity of any promoters constructs tested (data not shown). Cotransfecting hSRY expression constructs with rat RAS promoters significantly regulated each construct in the same manner as rat Sry [7] but with different relative intensities (Figure 3). Mutation of hSRY at amino acid 131 from proline to threonine (hSRY P131T) caused a significant increase in both *Ren* and *Ace2* promoter activities.

**Figure 4:**
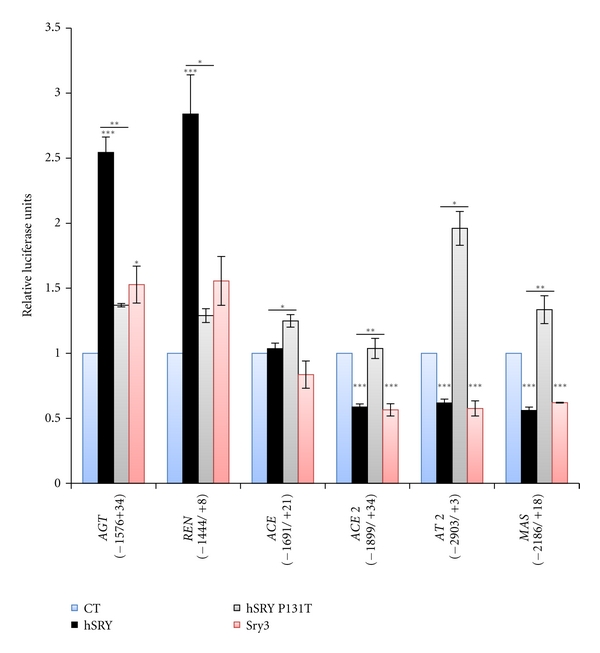
Human SRY regulation of the human RAS gene promoters. Human SRY (hSRY, black) significantly regulated human *AGT*, *REN, ACE 2, AT2*, and* MAS *promoters. Significance of hSRY is based on comparison to the control vector (blue). The mutation at amino acid 131 (hSRY P131T, gray) significantly diminished function in *AGT, REN*, *ACE 2, AT2*, and* MAS *constructs relative to hSRY. Sry3 (red) significantly regulated *AGT, ACE2, AT2*, and *MAS *compared to control (blue). *P ≤ 0.05, **P ≤ 0.01, ***P ≤ 0.001.

Human SRY significantly regulated the human promoters of *AGT *(*P* ≤ 0.001), *REN *(*P* ≤ 0.001), *ACE2 *(*P* ≤ 0.001), *AT2 *(*P* ≤ 0.001), and *MAS *(*P* ≤ 0.001) compared to control levels (Figure 4), elevating AGT and REN promoter activity while decreasing *ACE2*, *AT2*, and *MAS*. Mutation P131T caused an alteration in hSRY regulation on all these promoters. Sry3 significantly regulated *AGT, ACE2, AT2*, and *MAS*. The human ACE promoter construct used in these studies was not regulated by hSRY or Sry3.

## 4. Discussion

Hypertension is a complex disease, and distinguishing among a myriad of genetic and environmental factors is difficult. In humans and other animal models, blood pressure is higher in males than premenopausal females [14, 15]. This may be due to a genetic component that is male specific such as the Y-chromosome gene Sry. Accumulating evidence suggests that Sry has functions not directly related to testis determination [16]. This study, as well as others from our lab, suggests that Sry is a contributor to increasing blood pressure in SHR. Here we show that this Sry-mediated increase in blood pressure may translate into humans since both rat and human proteins seem to function similarly. Sry activation of the RAS gene promoters [7], combined with the effects of Sry on the sympathetic nervous system [8, 10], could contribute to sex differences in hypertension.

In SHR males, androgens have been shown to increase plasma Ren, renal Agt, and hepatic Agt [17]. Sry is known to interact with the androgen receptor (AR) and affect AR gene regulation [18]. It is thus probable that Sry and androgens coregulate the RAS and other genes.

In this study we have identified and characterized the amino acid in the rat Sry1 and Sry3 proteins that cause their differential effects on regulation of RAS gene promoters. Rat amino acid 38 (93 of human) has no published function or natural variation resulting in phenotypic change. Known structures of Sry and the models created here show that this amino acid is not likely to contribute to DNA interactions, and promoter activity assays support this.

The majority of variation of rat Sry protein differences on the RAS could be explained from results of mutations at amino acid 76 of rat (131 of human). In humans, a mutation of the proline to an arginine at amino acid 131 was present in an individual with sex reversal [19], showing significance of this amino acid. Modeling Sry1, Sry3, and hSRY suggests that the proline and threonine differences could account for altered interactions with DNA. NMR experiments showed that the proline of Sry interacts with the base pairs of the minor groove and the sugars of the DNA backbone [12]. Modeling variation through prediction of structure with iTASSER or changing structure 1j46 both showed a shift in the threonine of Sry3 to be more likely to interact with the phosphate backbone of DNA. The hinge region serves as a kinetic clamp for Sry-DNA interaction [20]. The hydrophobicity of the proline, at position 76/131, allows this residue to fit into the DNA minor groove, reducing the solvent accessible surface compared to the threonine (data not shown) and improving the stability of this Sry-DNA interaction. Alternatively, the presence of threonine may alter the solvent accessible surface and change the thermodynamics and kinetics of the Sry-DNA complex by potentially reducing kinetic clamp ability in Sry. In general, hSRY behaves similar to the rat Sry proteins with the exception that a change to threonine at amino acid 131 (correlating to rat amino acid 76) alters gene regulation from nonmutated much greater than in the rat. We believe that variations in the divergent bridge domain between rat and human may stabilize rat proteins containing a threonine, for example Sry3, more strongly than human SRY.

Although CHO cells are not normally used in analysis of the RAS, they provide us with several benefits. Co-transfection efficiency is very high in CHO cells. Also they are efficient for recombinant protein expression. Since CHO cells are from the ovary and lack the Y chromosome, there is no endogenous Sry expression. There is an ovarian tissue RAS, with ovary tissue or CHO cells shown to express prorenin, Ren, Ang, Ace, Ace 2, Ang II, and Ang II receptors [21–24]. Our promoter constructs contain only the proximal promoter region and a few hundred bases upstream, thus they cannot tell us if Sry activates RAS promoters differently *in vitro* or *in vivo*. Electroporation of Sry3 into the kidney increased Ang II and plasma Ren activity [9], which we believe is likely through the gene regulation we see in this study and thus provides *in vivo* gene support of the validity of our promoter activity assays.

We have long been interested in determining how human and mouse, with only a single *Sry* gene, can potentially perform all the functions of *Sry* encompassed by the multiple copies in rat. Human SRY behaves in a manner similar to both Sry1 and Sry3 in regulation of rat *Agt, Ren, Ace*, and *Ace2* promoters. Promoters of the human RAS showed similar responses to hSRY and Sry3, with hSRY P131T losing regulation. The human *ACE* promoter we used showed no significant change in activity with hSRY, and after analysis of Sry-binding sites using Genomatix MatInspector we could not identify any Sry-binding elements in the *ACE* promoter used in our experiments. Several potential Sry-binding sites are present in longer promoter sequences of human *ACE.* Additional experiments using longer *ACE* promoter sequences are necessary to address this issue, which will likely yield results similar to the rat *Ace* promoter. All other promoters used contained Sry and AP1-binding sites required for cis-Sry activation. In a previous study we showed that Sry acts primarily through the AP1 binding site for activation of the tyrosine hydroxylase promoter [10].

The data presented here suggest that human and rat Sry proteins and their genome targets (either cis or trans) regulating the RAS have remained functionally conserved. Regulation of the RAS by Sry appears to favor the production of Ang II, possibly reducing Ang-(1–7) and MAS levels [7]. MAS is known to be the receptor of Ang-(1–7) which can counter the effects of Ang II [25], thus the gene regulation by Sry suggests not only increasing the Ang II signaling, which has been shown *in vivo*, but also decreasing the counter pathway of Ang-(1–7). In addition, AT2 receptor promoter activity was decreased by human SRY, supporting its repressing effects on RAS components that oppose Ang II actions. Despite past uncertainty about the role of AT2 receptor, recent studies have confirmed AT2 receptor as a vasodilator mediator [26]. Human SRY likely plays a role similar to that of rat Sry in the regulation of blood pressure. It will be interesting to explore further the role this may serve in human hypertensive disease phenotypes.

## 5. Conclusions

Variations of amino acid 76 in rat (131 in human) led to differential regulation of rat RAS gene promoters. This study is the first to show that hSRY can regulate RAS promoters in the same pattern as rat Sry. In addition, by analyzing the human promoters of RAS, we show that potential cis- or trans-binding sites for Sry are conserved, indicating a conserved role of Sry in human blood pressure.

## Figures and Tables

**Table 1:** Sry expression constructs of rat and human showing the amino acid at 76 in rat or 131 in human.

Construct	76 (131)
Sry1	P
Sry1 P76T	T
Sry3	T
Sry3 T76P	P
hSRY	P
hSRY P131T	T

**Table 2:** Cloning of the human renin-angiotensin system gene promoters. The start and stop are the base pairs from the transcriptional start site of each gene.

Gene	Restriction enzymes	Right primer	Left primer	Size (bp)	Stop	Start
*AGT*	NcoI/SacI	ATACCATGGGGCCACTTCTGACCCTGCTG	GCCGAGCTCTAGAAGATCCCCCAGCTGATAG	1610	34	−1576
*REN*	NcoI/MluI	GTTCCATGGGAGGTTCTGTGGCTCCCTTAG	CGCACGCGTCTTCTTATGGGAAGCCCATTTA	1452	8	−1444
*ACE*	BglII/KpnI	CAGAGATCTGTGCTCGGCTCTGCCCCTTCTC	CGAGGTACCCCAAGCTGTTAGGACCCCTGAG	1700	21	−1691
*ACE 2*	HindIII/NheI	CCGAAGCTTTCCTGATCCTCTGTAGCCATGGGA	CGAGCTAGCAGGGCAGGCAGCATCTGACT	1932	34	−1899
*AT2r*	NcoI/MluI	CCGCCATGGGTCCACTGGGAGCCTTCAACCT	CGAACGCGTGGTGGAGGTGAGGCGGCAAA	2906	3	−2903
*Mas*	HindIII/NheI	CCGAAGCTTCCATGAGGAGGCCTCAGGTTGGA	CGAGCTAGCGCCCGTTTGGACCTGGTCGC	2204	18	−2186
